# Analysis of the Frequency and Associated Factors of Skin Toxicity in Patients Receiving Ribociclib-Based Therapy for Metastatic Breast Cancer

**DOI:** 10.3390/cancers18101602

**Published:** 2026-05-14

**Authors:** Esther Kim, Youra Lim, Ahrong Ham, Hyun Goo Kim, Jun Woo Lee, Jang Hee Lee, Joohyun Woo, Woosung Lim, Byung In Moon, Sei Hyun Ahn, Hye Ah Lee, Kyoung Eun Lee

**Affiliations:** 1 Department of Hematology and Oncology, Ewha Womans University Mokdong Hospital, Ewha Womans University, Seoul 07985, Republic of Korea; 41786@eumc.ac.kr (E.K.); 44203@eumc.ac.kr (Y.L.); aham@ewha.ac.kr (A.H.); 2 Department of Surgery, Ewha Womans University Mokdong Hospital, Ewha Womans University, Seoul 07985, Republic of Korea; kara8011@naver.com (H.G.K.); wnslee@ewha.ac.kr (J.W.L.); doctorlee85@ewha.ac.kr (J.H.L.); jwoo@ewha.ac.kr (J.W.); limw@ewha.ac.kr (W.L.); mbit@ewha.ac.kr (B.I.M.); ahnsh@amc.seoul.kr (S.H.A.); 3 Clinical Trial Center, Ewha Womans University Mokdong Hospital, Ewha Womans University, Seoul 07985, Republic of Korea; khyeah@ewha.ac.kr

**Keywords:** breast cancer, ribociclib, skin toxicity, metastatic breast cancer, CDK4/6 inhibitor, cutaneous adverse events

## Abstract

Introduction: In the treatment of hormone receptor-positive (HR+), HER2-negative (HER2−) metastatic breast cancer (MBC), the current guidelines recommend endocrine therapy combined with cyclin-dependent kinase 4/6 (CDK4/6) inhibitors as the preferred first-line treatment to preserve quality of life. Ribociclib is a CDK4/6 inhibitor that has been used in combination with aromatase inhibitors or fulvestrant in patients with HR+, HER2− metastatic breast cancer. Various adverse drug reactions associated with ribociclib have been reported, including cutaneous reactions, hepatotoxicity, and hematologic toxicity. In this study, we aimed to evaluate the clinical manifestations and risk factors of dermatologic toxicities in patients with metastatic breast cancer treated with ribociclib. Methods: This retrospective study included patients with metastatic/recurrent breast cancer who were prescribed ribociclib from April 2021 to December 2024 at a single institution. We retrospectively reviewed the medical records of these patients to identify the frequency of cutaneous adverse events, the time of onset, and the clinical characteristics of skin reactions. Logistic regression analysis was performed on several clinical factors, including body surface area (BSA) and concomitant medications, to identify risk factors associated with the occurrence of cutaneous adverse events. Results: A total of 110 patients with MBC were enrolled during the study period. The median age was 53 years (range, 28–82); all 110 patients (100.0%) were female; the median BSA was 1.56 m^2^ (range, 1.29–2.07); and 32 patients (29.1%) were premenopausal. Ribociclib plus letrozole was administered in 48 patients (43.6%) and ribociclib plus fulvestrant in 29 patients (26.4%). An additional 33 patients (30.0%) received ribociclib plus letrozole with a gonadotropin-releasing hormone (GnRH) agonist. Cutaneous adverse events occurred in 29 patients (26.4%), and the median time to onset was 84 days (range, 3–498). The cutaneous adverse event patterns included pruritus, erythematous macular rash, eczematous rash/contact dermatitis, vitiligo, urticarial reactions, polymorphous light eruption, toxic epidermal necrolysis (TEN), and desquamation. Grade 1 or 2 cutaneous adverse events occurred in 93.1% of patients; Grade 3 toxicity occurred in one patient; and Grade 4 toxicity, namely toxic epidermal necrolysis (TEN), was reported in one patient. Dose reduction was required in three patients (10.3%), and permanent discontinuation of ribociclib occurred in one patient. Clinical improvement was achieved in the majority of patients (86.2%) with cutaneous adverse events following supportive care. Logistic regression analysis revealed that age, Eastern Cooperative Oncology Group (ECOG) performance status, body surface area (BSA), treatment regimen, and use of cholesterol-lowering medications were not independently associated with the development of cutaneous adverse events. Conclusion: CDK4/6 inhibitors represent one of the most important treatment options for HR+/HER2− metastatic breast cancer. Regardless of their clinical efficacy, cutaneous adverse events remain a common source of patient discomfort. Therefore, careful clinical attention and appropriate supportive care are essential to improve patients’ quality of life.

## Simple Summary

Ribociclib, a cyclin-dependent kinase 4/6 (CDK4/6) inhibitor, is widely used in combination with endocrine therapy for hormone receptor-positive (HR+) and HER2-negative (HER2−) metastatic breast cancer. Although cutaneous adverse events (cAEs) are recognized side effects of CDK4/6 inhibitors, their incidence, clinical patterns, and associated risk factors in real-world practice remain incompletely characterized. In this retrospective, single-center study of 110 patients treated with ribociclib-based therapy, cAEs occurred in 26.4% of patients, with a median time to onset of 84 days. The most common manifestation was pruritus (58.6%), and the majority of events were mild to moderate in severity. No statistically significant risk factors for cAE development were identified. Antihistamines and topical agents were effective in most patients, and permanent treatment discontinuation was required in only one patient. These findings highlight the importance of early recognition and proactive multidisciplinary management of ribociclib-associated cutaneous toxicities to preserve treatment continuity and patients’ quality of life.

## 1. Introduction

Hormone receptor-positive (HR+)/HER2-negative (HER2−) metastatic breast cancer is one of the most common breast cancer subtypes worldwide. Recently, the treatment paradigm for HR+/HER2− metastatic breast cancer has changed significantly with the introduction of cyclin-dependent kinase 4/6 (CDK4/6) inhibitors [1]. Representative agents include palbociclib, ribociclib, and abemaciclib, which exert anticancer effects by blocking cell cycle progression when used in combination with endocrine therapy. The therapeutic efficacy of ribociclib has been firmly established through the phase III MONALEESA-2, -3, and -7 clinical trials, demonstrating significant improvements in progression-free survival (PFS) and overall survival (OS) in both pre- and postmenopausal women when used in combination with endocrine therapy [2,3,4,5].

However, despite these therapeutic benefits, CDK4/6 inhibitors are associated with various adverse events, with cutaneous adverse events (cAEs) being among the more frequent side effects that are often overlooked yet carry significant clinical impact [6,7,8,9,10]. The spectrum of skin toxicities caused by CDK4/6 inhibitors is diverse, ranging from relatively common manifestations, such as pruritus, eczematous dermatitis, maculopapular rash, urticarial reactions, and xerosis, to serious and rare reactions, such as vitiligo-like lesions, cutaneous lupus erythematosus, bullous dermatitis, and toxic epidermal necrolysis (TEN) [11,12,13,14,15,16,17,18,19,20,21]. Furthermore, several recent studies have reported additional cutaneous toxicities when ribociclib or other CDK4/6 inhibitors are used concurrently with radiotherapy, including enhanced radiation dermatitis and radiation recall reactions, raising concerns about the safety of this combination in clinical practice [22,23].

The underlying mechanisms of ribociclib-associated cAEs are diverse and vary according to the specific type of skin reaction. In a rat model, based on the ribociclib mechanism that inhibits CDK4/6 activity by competitively binding to adenosine 5′-triphosphate (ATP) binding sites, the suppression of ATP has been proposed as a possible contributor to skin damage [24].

With regard to vitiligo-like lesions, a growing number of case reports have been published suggesting that the pathogenesis is deeply associated with immune-mediated mechanisms, including the destruction of melanocytes driven by an autoimmune process, as supported by histopathological findings such as lymphocytic infiltration along the basement membrane layer [12,25].

Therefore, in this study, our aim is to evaluate the incidence, characteristics, and clinically associated factors of cAEs, as well as the effects on survival outcomes, in patients with metastatic and recurrent breast cancer receiving ribociclib-based treatment.

## 2. Materials and Methods

### 2.1. Study Design and Patients

In this retrospective, noninterventional study, the clinical characteristics of skin adverse events associated with ribociclib-based therapy in HR+/HER2− metastatic breast cancer were evaluated in a single-center, real-world setting. The inclusion criteria were as follows: (1) patients diagnosed with HR+/HER2− metastatic or recurrent breast cancer; (2) patients who received at least one dose of a ribociclib-based regimen between April 2021 and December 2024 at our institution; and (3) patients with available and complete medical records. The exclusion criteria were as follows: (1) patients with concurrent malignancies other than breast cancer at the time of ribociclib initiation; (2) patients with insufficient medical records for data extraction; and (3) patients enrolled in other interventional clinical trials during the same treatment period. The patients’ baseline characteristics at ribociclib administration, including comorbidities, body surface area (BSA), skin reactions, and toxicity grade, were collected through a retrospective review of medical records.

The trial was performed in accordance with the standards of Good Clinical Practice and the Declaration of Helsinki. The trial protocol was approved by the Institutional Review Board (IRB) of our institution (IRB No. EUMC 2025-04-023).

### 2.2. Statistical Analysis

The demographics and clinical characteristics of the patients were analyzed using descriptive statistics. Continuous variables were presented as means with standard deviations or medians with ranges, while categorical variables were presented as frequencies and percentages. To identify risk factors related to cAEs, logistic regression analysis was performed, and the associations were expressed as odds ratios (ORs) with 95% confidence intervals (95% CIs). Variables satisfying *p* < 0.2 in univariate analysis were considered in the multivariate model. Additionally, we evaluated the association between the presence of cAEs and disease progression using Kaplan–Meier analysis and the log-rank test. We further used Cox proportional hazards models to assess the association of cAEs and related factors with disease progression according to the treatment regimen, and the results were reported as hazard ratios (HRs) with 95% CIs. The proportional hazards assumption was assessed using the Schoenfeld residuals test and was found to be satisfied. All *p*-values were two-tailed, and a *p*-value < 0.05 was considered statistically significant. All statistical analyses were performed using SAS version 9.4 (SAS Institute, Cary, NC, USA) and R version 4.5.1 (R Foundation for Statistical Computing, Vienna, Austria).

## 3. Results

### 3.1. Patient Characteristics

**Figure 1:**
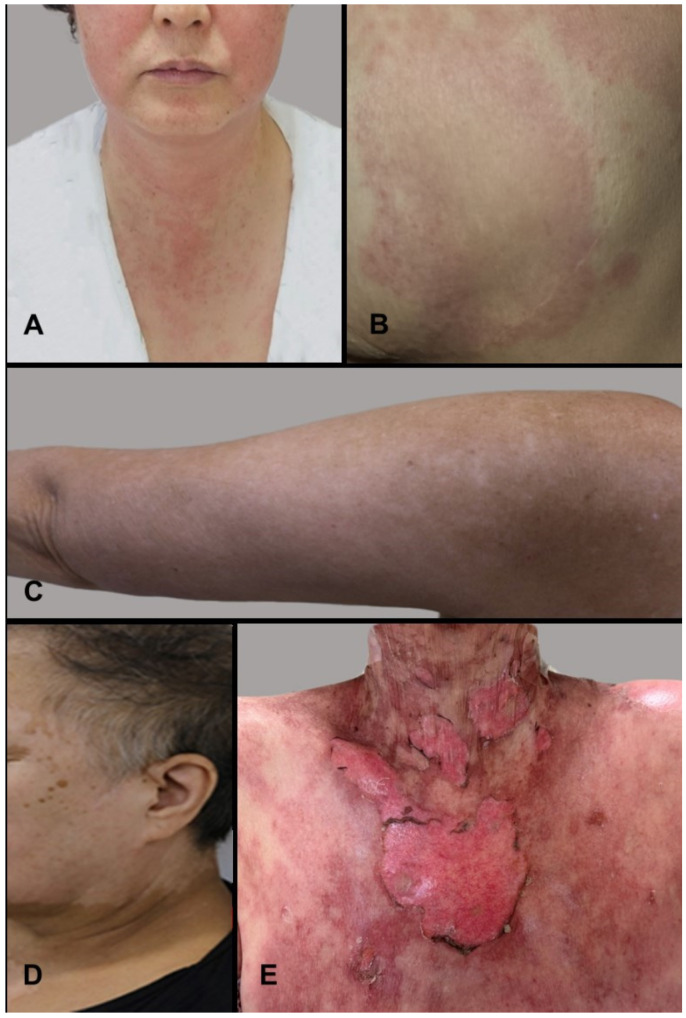
Representative clinical photographs of cutaneous AEs observed in patients receiving ribociclib-based therapy. (**A**): erythematous rash on the face and anterior neck; (**B**): eczematous patch on the left chest; (**C**): vitiligo-like hypopigmented macules on the left triceps; (**D**): vitiligo-like hypopigmented macules on the lateral face; (**E**): severe exfoliative dermatitis with necrosis (TEN).

The characteristics of the 110 enrolled patients are presented in Table 1. The median follow-up duration was 13.3 months, and the median age was 53 years (range, 28–82). The number of patients with an Eastern Cooperative Oncology Group (ECOG) performance status (PS) of 0 or 1 was 107 (97.3%), and 78 patients (70.9%) were postmenopausal. The median BSA was 1.56 m^2^ (range, 1.29–2.07), and the mean BSAs when classified into tertiles were 1.45 (±0.06), 1.57 (±0.04), and 1.74 (±0.10), respectively. Forty-eight patients (43.6%) received ribociclib + letrozole treatment, 29 (26.4%) patients received ribociclib + fulvestrant, and 33 (30.0%) patients received ribociclib + letrozole + GnRH agonist. Hypercholesterolemia was present in 39 patients (36.4%), and 19 patients (17.3%) were receiving cholesterol-lowering medications at the time of treatment initiation. Cutaneous adverse events were observed in 29 of 110 patients (26.4%). Among these, the majority presented with mild to moderate reactions—Grade 1 toxicity was the most frequent (17 patients, 58.6%), followed by Grade 2 (10 patients, 34.5%), while Grade 3 and Grade 4 toxicities each occurred in one patient (3.5% each)—and the median time to skin reaction appearance was 84 days (range, 3–498). The most common dermatologic manifestation was pruritus, affecting 17 patients (58.6%), followed by erythromacular rash in eight patients (27.6%), eczematous rash or contact dermatitis in four patients (13.8%), vitiligo and urticarial-type reactions each in three patients (10.3%), polymorphous light eruption in two patients (6.9%), and toxic epidermal necrolysis (TEN) and desquamation each in one patient (3.4%) (Table 1 and Figure 1).

### 3.2. Factors Associated with Skin Toxicity Occurrence

Logistic regression analyses were performed to identify clinical predictors of cAEs. In the univariate analysis, no statistically significant association was identified between cAEs and age (OR 2.61, 95% CI 0.73–9.30; *p* = 0.140), menopausal status (*p* = 0.495), ECOG performance status (*p* = 0.585), BSA (OR 11.13, 95% CI 0.55–226.29; *p* = 0.117), treatment regimen, disease presentation, hypercholesterolemia (*p* = 0.274), or use of cholesterol-lowering medications (OR 2.42, 95% CI 0.86–6.81; *p* = 0.093). In the multivariate analysis, none of the evaluated variables reached statistical significance, including age (OR 1.75, 95% CI 0.43–7.06; *p* = 0.435), use of cholesterol-lowering medications (OR 2.38, 95% CI 0.79–7.13; *p* = 0.122), and BSA (OR 5.85, 95% CI 0.24–140.94; *p* = 0.277) (Table 2).

### 3.3. Clinical Impacts of Skin Toxicities

Among the 29 patients who developed cAEs, the median time to onset of skin reaction was 84 days (range, 3–498 days). Ribociclib dose reduction due to cAEs was required in three patients (10.3%), while 26 patients (89.7%) continued the treatment without dose modification. Treatment interruption or discontinuation due to skin toxicity occurred in three patients (10.3%). Of these, one patient permanently discontinued ribociclib due to a Grade 4 cutaneous adverse event, while the remaining two patients underwent temporary treatment interruptions of one to two weeks, after which ribociclib was successfully resumed and continued without the recurrence of severe toxicity. For the management of dermatologic adverse events, antihistamines were the most frequently used agent (10 patients, 35.7%), followed by topical ointments (eight patients, 28.6%), other medications (seven patients, 25.0%), and systemic corticosteroids (three patients, 10.7%) (Table 3).

### 3.4. Disease Progression and Cutaneous Toxicities

**Figure 2:**
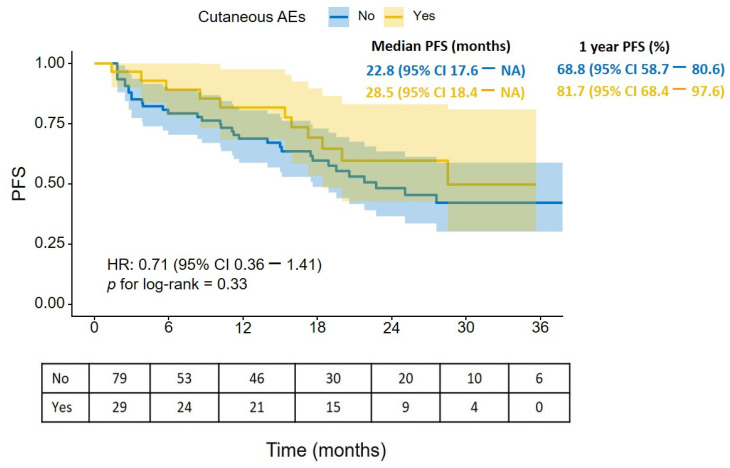
PFS according to the presence of cutaneous adverse events. Kaplan–Meier estimates of PFS are shown for patients who developed cutaneous adverse events (cAEs; *n* = 29, yellow) and those who did not (*n* = 79, blue). Shaded areas represent 95% CIs. *p* value was calculated using the log-rank test. NA, not reached (the upper bound of the 95% confidence interval had not been attained by the end of the observation period).

Kaplan–Meier analysis demonstrated a numerically longer PFS in patients who developed cAEs compared with those who did not (median PFS 28.5 months, 95% CI 18.4–NA vs. 22.8 months, 95% CI 17.6–NA). The 1-year PFS rate was also higher in the cAE group (81.7%, 95% CI 68.4–97.6) than in the non-cAE group (68.8%, 95% CI 58.7–80.6). However, this difference did not reach statistical significance (HR 0.71, 95% CI 0.36–1.41; log-rank *p* = 0.33), suggesting a trend toward improved PFS in patients experiencing cAEs, although the current sample size may have been insufficient to detect a statistically significant difference (Figure 2).

Cox proportional hazards regression analysis revealed no statistically significant association between disease progression and any of the evaluated variables—including cAEs, age (≥65 years), BSA, disease status, and cholesterol-lowering medication use—across either the ribociclib plus letrozole with GnRH agonist or ribociclib plus fulvestrant regimens, indicating that none of the examined variables independently predicted disease progression regardless of treatment regimen (Table 4).

## 4. Discussion

In the pivotal phase III MONALEESA-2, -3, and -7 trials, which represent the cornerstone evidence for ribociclib in the treatment of metastatic and recurrent breast cancer, cutaneous adverse events (cAEs) were reported in 13% to 22% of patients [2,4,5,8,26]. In the present study, the incidence of cAEs was 26.4%, which is somewhat higher than that observed in these prospective trials. Several factors may account for this discrepancy. In the pivotal phase III trials, mild symptoms such as pruritus may not have been consistently captured or documented, potentially leading to an underestimation of the true incidence. Furthermore, in real-world clinical settings, polypharmacy and other patient-related factors may contribute to a higher rate of cAEs compared with the controlled conditions of clinical trials. Notably, the cAEs reported in the pivotal trials were largely described in nonspecific terms—predominantly as mild rash or pruritus—with insufficient subtype classification. The present study addresses this limitation by systematically characterizing the specific types, time of onset, and clinical outcomes of ribociclib-associated cutaneous toxicities in a real-world setting, thereby providing a more granular analysis than was available from the pivotal trial data.

Cutaneous adverse events associated with anticancer agents can arise through diverse pathophysiological mechanisms depending on the specific drug class [27]. Classic cytotoxic chemotherapy induces direct damage to rapidly dividing skin cells by inhibiting DNA replication. More recently, targeted agents—particularly epidermal growth factor receptor (EGFR) tyrosine kinase inhibitors—are well known to cause acneiform eruptions, xerosis, and pruritus as characteristic dermatologic toxicities. In contrast, CDK4/6 inhibitors have been associated with a broader range of proposed mechanisms, including intracellular ATP suppression, increased oxidative stress, immune-mediated reactions, and photosensitivity [15,24,28,29]. Compared with other anticancer drug classes, CDK4/6 inhibitor-induced cAEs are notably diverse and encompass a wide clinical spectrum, ranging from mild and nonspecific reactions to rare but potentially life-threatening conditions.

In addition to the established mechanisms, emerging evidence suggests that cross-talk between sex hormone receptor signaling and CDK4/6 pathways may contribute to cAE development. Estrogen and progesterone receptors are expressed not only in breast tumor cells but also in keratinocytes and dermal fibroblasts, where they regulate skin homeostasis, barrier function, and wound healing. CDK4/6 inhibition disrupts the cell cycle in these skin cells, and this effect may be further amplified by the concurrent suppression of estrogen signaling through aromatase inhibitors or fulvestrant. This dual interference with both hormone receptor signaling and CDK4/6-driven cell cycle progression in skin tissue may partly underlie the relatively high frequency and diverse clinical spectrum of cAEs observed in patients receiving ribociclib-based combination therapy, as compared with CDK4/6 inhibitor monotherapy [30].

Historically, anticancer drug dosing has been based on body surface area (BSA), under the assumption that patients with a larger body size have a greater volume of drug distribution and metabolic capacity, and therefore require higher doses. However, accumulating evidence suggests that BSA may not reliably predict pharmacokinetic parameters for many agents, and a substantial proportion of recently developed targeted therapies have been introduced with fixed-dose regimens [31].

Ribociclib is administered at a fixed dose of 600 mg per day, without BSA-based dose adjustment. Although the present study examined the potential relationship between BSA and the occurrence of cAEs, no statistically significant association was identified.

Another clinically relevant consideration is the potential pharmacokinetic interaction between ribociclib and cholesterol-lowering medications, particularly statins. Ribociclib is primarily metabolized via cytochrome P450 3A4 (CYP3A4), and concomitant use with statins that share this metabolic pathway—such as simvastatin and lovastatin—may result in elevated systemic statin concentrations, potentially increasing the risk of statin-induced myopathy. In our cohort, 19 patients (17.3%) were receiving cholesterol-lowering medications at the time of ribociclib initiation. Although systematic monitoring of serum creatinine kinase (CK) levels and renal function as markers of statin-related toxicity was not a predefined endpoint of this retrospective study, no clinically significant musculoskeletal adverse events attributable to a drug–drug interaction were documented in this subgroup during the observation period. Nonetheless, clinicians should be vigilant regarding this interaction and may consider monitoring CK levels and renal function in patients receiving ribociclib concurrently with CYP3A4-metabolized statins, particularly in those with predisposing risk factors [32,33].

In the present study, although the development of cAEs did not demonstrate a statistically significant impact on PFS, a numerical trend toward superior PFS was observed in patients who experienced cAEs compared with those who did not. Given that immune-mediated mechanisms have been proposed as one of the pathways underlying CDK4/6 inhibitor-associated cAEs, it is conceivable that the relationship between cutaneous toxicity and disease control may differ in the context of combination regimens incorporating immune checkpoint inhibitors. Future studies investigating the clinical outcomes and underlying mechanisms of cAEs in such combination settings are warranted.

Regarding the use of systemic corticosteroids in the management of cAEs, three patients (10.7%) in our cohort received systemic glucocorticoids. While glucocorticoids are frequently employed in oncology to attenuate treatment-related side effects, preclinical studies have raised concerns that systemic glucocorticoid exposure may promote disease progression in certain aggressive breast cancer subtypes by facilitating glucocorticoid receptor-mediated signaling that circumvents endocrine therapy. However, in the context of HR+/HER2− MBC, the clinical relevance of short-term, low-to-moderate-dose glucocorticoid use for cAE management has not been definitively established. In the present study, corticosteroids were prescribed for short-course treatment of severe or refractory skin reactions, and the anticipated clinical benefit of controlling significant cAEs was judged to outweigh the theoretical oncologic risk. The risk–benefit ratio of systemic corticosteroid use should be evaluated on an individual patient basis, and prolonged or high-dose regimens should be avoided in the absence of a compelling clinical indication [34].

Cutaneous adverse events occurring during ribociclib therapy can cause considerable discomfort in daily life and may lead to reduced treatment adherence, potentially compromising therapeutic efficacy. Therefore, early recognition and proactive management of skin reactions through a multidisciplinary approach are essential components of breast cancer care. Clinicians involved in the management of patients receiving ribociclib should maintain a high level of awareness regarding the full spectrum of associated cutaneous toxicities and their appropriate management strategies.

The present study has several limitations that should be acknowledged. First, the retrospective, single-center design may introduce selection bias and limit the generalizability of the results to broader or more diverse patient populations. Second, the relatively small sample size (n = 110) may have constrained the study’s statistical power to detect significant associations between clinical variables and cAE occurrence or severity. Third, the retrospective ascertainment of cAEs relied on clinical documentation, which may be subject to underreporting, particularly for mild or transient symptoms such as pruritus. Fourth, patient-reported outcome measures and quality-of-life data were not systematically collected, limiting a comprehensive evaluation of the subjective burden imposed by cAEs on patients. Future prospective, multicenter studies incorporating standardized cAE assessment instruments, patient-reported outcome measures, and mechanistic analyses are needed to validate these findings and to develop evidence-based clinical guidelines for the prevention and management of ribociclib-associated cutaneous toxicities.

## 5. Conclusions

The use of CDK4/6 inhibitors has now become an integral component of breast cancer management, and the selection of an appropriate agent must be guided by each individual patient’s clinical status and the distinct adverse event profile of each drug. In particular, cutaneous toxicities require a multidisciplinary approach involving systematic patient care, wherein the oncologist plays a pivotal role in mitigating long-term treatment-related toxicities and improving the patient’s quality of life.

## Figures and Tables

**Table 1:** Patient characteristics and cutaneous adverse events (*N* = 110).

	Number of Patients			
	Missing Value	Analyzed Patients	*N* (%)	Mean (±S.D) or Median (Range)
Age, years	0	110		53.61 (±10.43)
Median (range)				53.00 (28.00–82.00)
Sex	0	110		
Female			110 (100.0%)	
Male			0 (0.0%)	
Menopausal status	0	110		
No			32 (29.1%)	
Yes			78 (70.9%)	
ECOG performance status	0	110		
0			77 (70.0%)	
1			30 (27.3%)	
2			3 (2.7%)	
BSA (m^2^)				1.56 (1.29–2.07)
Tertile 1 (<1.51)			36 (33.0%)	1.45 (±0.06)
Tertile 2 (≥1.51 to <1.64)			37 (33.9%)	1.57 (±0.04)
Tertile 3 (≥1.64)			36 (33.0%)	1.74 (±0.10)
Treatment	0	110		
Ribociclib + letrozole			48 (43.6%)	
Ribociclib + fulvestrant			29 (26.4%)	
Ribociclib + letrozole + GnRHa			33 (30.0%)	
Disease status				
De novo metastatic	0	110	45 (40.9%)	
Recurrent			65 (59.1%)	
Hypercholesterolemia	3	107		
No			68 (63.6%)	
Yes			39 (36.4%)	
Taking cholesterol-lowering medication				
No	0	110	91 (82.7%)	
Yes			19 (17.3%)	
Cutaneous adverse events	0	110		
No			81 (73.6%)	
Yes			29 (26.4%)	
Toxicity grade (CTCAE)				
1	0	29	17 (58.6%)	
2			10 (34.5%)	
3			1 (3.5%)	
4			1 (3.5%)	
Patterns of skin toxicity	0	29		
Pruritus			17 (58.6%)	
Erythematous macular rash			8 (27.6%)	
Eczematous rash/contact dermatitis			4 (13.8%)	
Vitiligo-like lesion			3 (10.3%)	
Urticarial-type			3 (10.3%)	
Polymorphous light eruption			2 (6.9%)	
TEN			1 (3.4%)	
Desquamation			1 (3.4%)	

S.D, standard deviation; ECOG, Eastern Cooperative Oncology Group; BSA, body surface area; CTCAE, Common Terminology Criteria for Adverse Events; GnRHa, GnRH agonist.

**Table 2:** Assessment of risk factors associated with cutaneous adverse events.

	Univariate		Multivariate	
	OR (95% CI)	*p* Value	OR (95% CI)	*p* Value
Age, years				
<65 years	Ref		Ref	
≥65 years	2.61 (0.73–9.30)	0.140	1.75 (0.43–7.06)	0.435
Menopausal status				
No	Ref			
Yes	1.40 (0.53–3.71)	0.495		
ECOG performance status				
0	Ref			
1	0.76 (0.29–2.03)	0.585		
2	-	-		
BSA (m^2^)	11.13 (0.55–226.29)	0.117	5.85 (0.24–140.94)	0.277
Tertile 1 (<1.51)	Ref			
Tertile 2 (≥1.51 to <1.64)	1.30 (0.45–3.78)	0.634		
Tertile 3 (≥1.64)	1.54 (0.53–4.44)	0.424		
Treatment				
Ribociclib + letrozole	Ref		Ref	
Ribociclib + fulvestrant	0.38 (0.12–1.18)	0.093	0.49 (0.14–1.67)	0.252
Ribociclib + letrozole + GnRHa	0.49 (0.18–1.37)	0.173	0.62 (0.20–1.90)	0.398
Disease status				
De novo metastatic	Ref		Ref	
Recurrent	0.55 (0.23–1.29)	0.170	0.56 (0.21–1.45)	0.230
Hypercholesterolemia				
No	Ref			
Yes	1.63 (0.68–3.88)	0.274		
Taking cholesterol-lowering medication				
No	Ref		Ref	
Yes	2.42 (0.86–6.81)	0.093	2.38 (0.79–7.13)	0.122

OR, odds ratio; 95% CI, 95% confidence interval; ECOG, Eastern Cooperative Oncology Group; BSA, body surface area.

**Table 3:** Clinical course in patients with cutaneous adverse events (*n* = 29).

	Number of Patients			
	Missing Value	Analyzed Patients	*N* (%)	Mean (±S.D) or Median (Range)
Time until cAEs appear (days)	1	28		
Median (range)				84.00 (3.00–498.00)
Dose reduction due to skin toxicity	0	29		
No			26 (89.7%)	
Yes			3 (10.3%)	
Drug discontinuation due to skin toxicity	0	29		
No			26 (89.7%)	
Yes *			3 (10.3%)	
Medications used for cAEs	1	28		
Antihistamine			10 (35.7%)	
Topical ointment			8 (28.6%)	
Others			7 (25.0%)	
Systemic steroid			3 (10.7%)	

AEs, adverse events; cAEs, cutaneous adverse events; S.D, standard deviation. * One patient permanently discontinued ribociclib due to Grade 4 toxicity; two patients resumed treatment after a 1–2-week interruption.

**Table 4:** Factors associated with progression according to treatment regimen.

	Treatment Patterns			
	Ribociclib + Letrozole ± GnRHa		Ribociclib + Fulvestrant	
	HR (95% CI)	*p* Value	HR (95% CI)	*p* Value
Cutaneous AEs (yes)	0.67 (0.27–1.67)	0.393	0.75 (0.22–2.59)	0.652
Age (≥65 years)	0.57 (0.13–2.54)	0.457	1.78 (0.36–8.88)	0.484
BSA (m^2^)	4.85 (0.36–66.18)	0.236	9.81 (0.19–504.57)	0.256
Disease status (recurrent)	0.97 (0.44–2.15)	0.944	0.85 (0.18–4.04)	0.841
Taking cholesterol-lowering medication (yes)	1.03 (0.38–2.76)	0.955	1.32 (0.42–4.20)	0.637

HR, hazard ratio; 95% CI, 95% confidence interval; BSA, body surface area. Hazard ratios and 95% confidence intervals were estimated from a multivariable Cox proportional hazards model adjusted for all variables listed in the table.

## Data Availability

The data presented in this study are not publicly available due to patient privacy considerations. Data are available from the corresponding author upon reasonable request and with appropriate ethical approval.

## Abbreviations

BSA: body surface area; cAE: cutaneous adverse event; CDK4/6: cyclin-dependent kinase 4 and 6; CI: confidence interval; CK: creatinine kinase; CTCAE: Common Terminology Criteria for Adverse Events; ECOG PS: Eastern Cooperative Oncology Group performance status; GnRH: gonadotropin-releasing hormone; GnRHa: GnRH agonist; HER2: Human Epidermal Growth Factor Receptor 2; HR: hazard ratio/hormone receptor; HR+: hormone receptor-positive; HER2−: HER2-negative; MBC: metastatic breast cancer; OR: odds ratio; OS: overall survival; PFS: progression-free survival; TEN: toxic epidermal necrolysis.
